# Clot reduction prior to embolectomy: mSAVE as a first-line technique for large clots

**DOI:** 10.1371/journal.pone.0216258

**Published:** 2019-05-09

**Authors:** Marios-Nikos Psychogios, Ioannis Tsogkas, Alex Brehm, Amelie Hesse, Ryan McTaggart, Mayank Goyal, Ilko Maier, Marlena Schnieder, Daniel Behme, Volker Maus

**Affiliations:** 1 Department of Neuroradiology, University Medical Center Goettingen, Goettingen, Germany; 2 Department of Neurology, University Medical Center Goettingen, Goettingen, Germany; 3 Department of Radiology, Neurology, and Neurosurgery, Warren Alpert Medical School of Brown University, Providence, Rhode Island, United States of America; 4 Calgary Stroke Program, Department of Clinical Neurosciences, University of Calgary, Calgary, Alberta, Canada; 5 Department of Neuroradiology, Clinic for Radiology & Nuclear Medicine, University Hospital Basel, Basel, Switzerland; 6 Department of Radiology, Neuroradiology and Nuclear Medicine, Ruhr University Bochum, Knappschaftskrankenhaus Bochum, Bochum, Germany; University of Münster, GERMANY

## Abstract

**Introduction:**

The “Stent retriever Assisted Vacuum-locked Extraction” (SAVE) technique is a promising embolectomy method for intracranial large vessel occlusion (LVO). We report our experience using a modified SAVE (mSAVE) approach for clot reduction prior to embolectomy in acute ischemic stroke patients with large clots.

**Materials and methods:**

We retrospectively analyzed 20 consecutive patients undergoing mSAVE in our center due to intracranial LVO. Angiographic data (including first-pass and overall complete reperfusion, defined as an expanded Thrombolysis in Cerebral Infarction (eTICI) score of 3, rate of successful reperfusion (eTICI ≥2c), number of passes, time from groin puncture to reperfusion) and clinical data (favorable outcome at 90 days, defined as modified Rankin Scale (mRS) ≤2) were assessed.

**Results:**

First-pass and overall eTICI 3 reperfusion was reached in 13/20 (65%) and 14/20 (70%), respectively. The rate of successful reperfusion (eTICI ≥2c) after one pass was 85% and on final angiogram 90% with an average number of 1.1 ± 0.3 attempts. Eight out of 11 (73%) ICA occlusions were reperfused successfully and 5 (46%) completely after a single pass. Median groin to reperfusion time was 33 minutes (IQR 25–46). A favorable clinical outcome was achieved in 9/20 (45%) patients at discharge and after 90 days, respectively.

**Conclusion:**

Clot reduction followed by embolectomy (mSAVE) is feasible and may be an important tool in the treatment of large clots.

## Introduction

One of the major predictors of a favorable outcome in patients with acute intracranial large vessel occlusion (LVO) is complete and swift reperfusion of the affected territory [1]. Embolectomy in combination with intravenous thrombolysis (IVT) has been the state-of-the-art therapy for acute ischemic stroke (AIS) since 2015, after several randomized controlled trials (RCTs) demonstrated that endovascular treatment of LVO using stent retrievers is more effective than IVT alone [2–4]. Recent studies indicated that reperfusion success might be influenced by several factors. In a post hoc analysis of the Contact Aspiration vs Stent Retriever for Successful Revascularization (ASTER) trial it was shown that successful reperfusion was less frequently achieved in patients with high clot burden [5]. Furthermore, a sub-analysis of the North American Solitaire Acute Stroke (NASA) Registry revealed a lower first-pass effect and more embolectomy maneuvers in patients with internal carotid artery (ICA) terminus occlusions, which is known to be associated with a large clot amount [6,7]. As the probability of successful reperfusion and favorable clinical outcome decreases with the number of passes and prolonged procedure time, our aim was to develop an embolectomy technique which is able to solve the problem of large clot burden [8]. Therefore, we modified the recently published “Stent retriever Assisted Vacuum-locked Extraction” (SAVE) technique to increase the probability of reperfusion success and decrease the number of retrieval maneuvers by reducing the amount of clot prior to embolectomy [9]. We here present our first experience with the modified SAVE (“mSAVE”) technique in AIS patients.

## Materials and methods

### Study design and patient selection

A retrospective analysis was performed to identify all patients who underwent embolectomy with SAVE at our hospital between November 2015 and March 2018. Of those, only patients who were treated with mSAVE were included for further analysis. The decision of performing mSAVE during the intervention was left to the attending neuroradiologist. Baseline, clinical, and angiographic parameters were extracted from a prospectively acquired database. The extent of thrombus amount was evaluated with the clot burden score (CBS) based on computed tomography angiography (CTA) [10]. Clot length was measured on axial and coronal reconstructed non-contrast CT images with ICA-MCA length in cases of distal ICA occlusions. The National Institute of Health Stroke Scale (NIHSS) parameter and the modified Rankin Scale (mRS) at discharge and after 90 days was assessed by a certified stroke neurologist. Inclusion criteria were evidence of an intracranial LVO diagnosed on multi-detector non-contrast CT and CTA or on FDCT/FDCTA in cases of one-stop management and the intention to perform endovascular stroke treatment [11]. All eligible patients received IVT according to the guidelines of the National Neurological Society. Non-contrast CT or magnet resonance imaging was regularly performed within 24 hours after treatment, or immediately in a symptomatic patient. Symptomatic intracranial hemorrhage (sICH) was defined as any extravascular blood in the brain or within the cranium that was associated with clinical deterioration, as defined by an increase of ≥ 4 points in the NIHSS score.

First-pass and overall complete reperfusion was defined as an expanded Thrombolysis in Cerebral Infarction (eTICI) score of 3. The rate of successful reperfusion was defined as eTICI ≥2c. Furthermore, the number of passes and time from groin puncture to reperfusion was assessed. A favorable outcome at 90 days was defined as mRS ≤2.

All images from digital subtraction angiography were re-evaluated in accordance with the recommendations of the Cerebral Angiographic Revascularization Grading (CARG) Collaborators from an internal core-team that was not involved in the evaluated procedures. The core-team was blinded to the clinical outcome, presentation, or any demographic data to assess pre-interventional, initial state and extent of tissue reperfusion after each embolectomy attempt. According to the guidelines of the local ethics committee, ethic approval was given for this retrospective study from the institutional review board of the University Medical Center Goettingen (No: 13/7/15An), which was conducted in accordance to the Declaration of Helsinki. Written patient consent was obtained.

### Endovascular treatment with mSAVE

**Fig 1:**
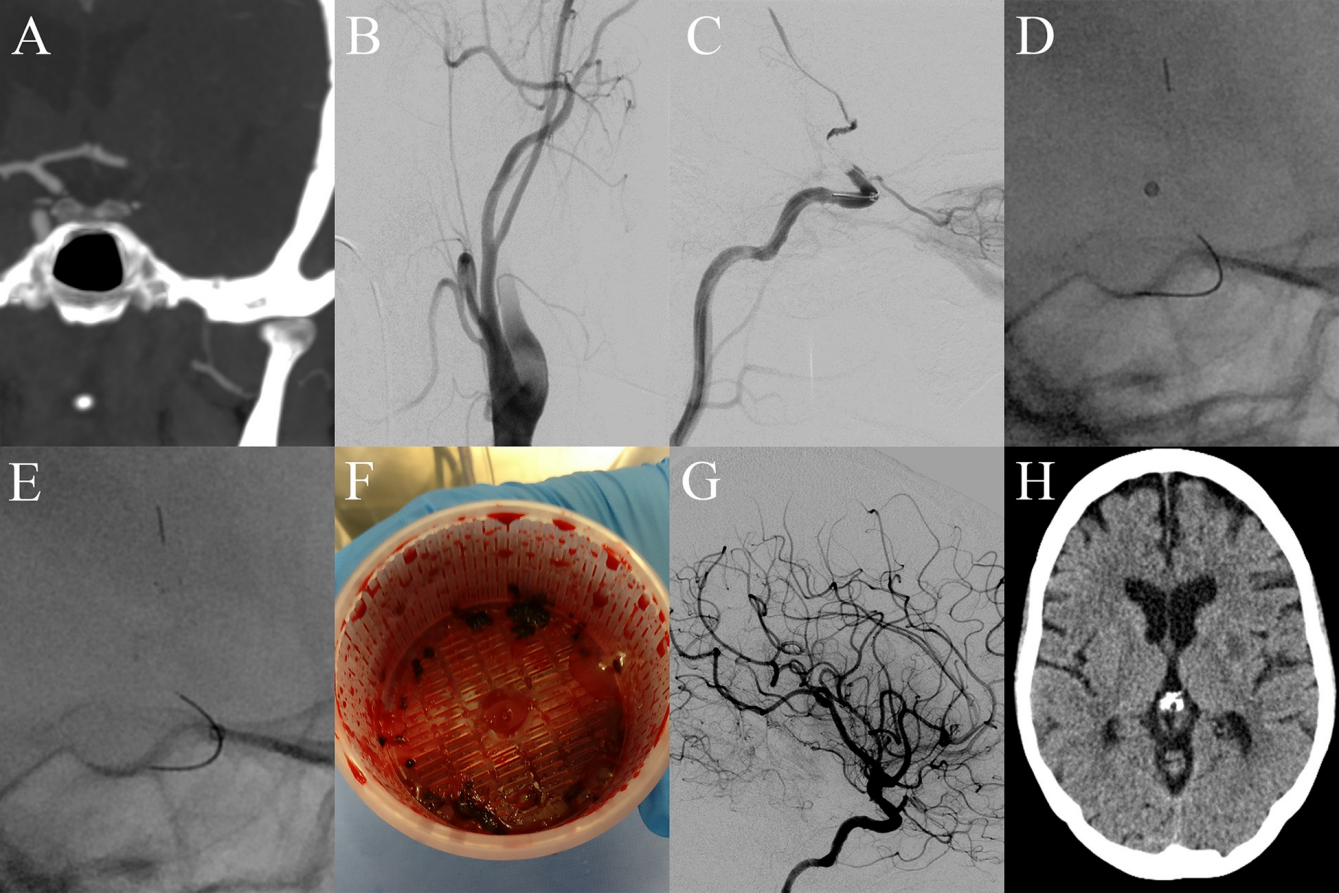
Endovascular treatment with mSAVE. A, CT-angiography (coronal view) of a patient suffering from a distal occlusion of the internal carotid artery (ICA) on the left site. B, First angiogram demonstrating extracranial pseudo-occlusion of the ICA. C, Positioning of a stent retriever in the distal ICA and proximal M1 segment with antegrade flow via the deployed stent retriever. D, The aspiration catheter is connected to an activated pump and advanced to the proximal face of the clot until vacuum is achieved. E, The aspiration catheter is then retrieved along the wire of the stent retriever without moving the stent retriever itself in order to reduce thrombus mass and subsequently advanced to the occlusion site after vacuum is lost. F, The pump canister is showing massive amount of clot prior to embolectomy. G, After performing one attempt of SAVE maneuver the intracranial lesion is recanalized and the downstream territory completely reperfused. H, Final computed tomography at discharge demonstrating a small infarction within the basal ganglia.

SAVE has been recently described [9]. Based on a triaxial approach, an 8 French (F) balloon-guide catheter (Flowgate^2^ Stryker Neurovascular, Fremont, CA, USA) or a guide catheter (8F Cordis Vista, Johnson & Johnson, New Brunswick, NY, USA) is placed distally in the internal carotid artery (ICA) or in cases of a posterior circulation stroke a 7F guide catheter (Mach 1, Boston Scientific, Marlborough, MA, USA) within the vertebral artery. A microcatheter is advanced through a 5F/6F aspiration catheter (AXS Catalyst, Stryker Neurovascular; Sofia/Sofia Plus, Microvention, Tustin, CA, USA) past the occlusion site under continuous aspiration or with the help of a 0.014ˈˈ inch microwire. A stent retriever (Trevo variants, Stryker Neurovascular; EmboTrap, Neuravi, Galway, Irland) is placed primarily distally to and with the proximal third across the occlusion site by using the active push deployment technique [12]. The microcatheter is slowly retracted in order to maximize the inner volume of the aspiration catheter. We then connect the aspiration catheter to an aspiration pump and advance to the proximal face of the clot in order to achieve dynamic vacuum. In our modified approach, the aspiration catheter is then retrieved along the wire of the stent retriever without moving the stent retriever itself in order to reduce thrombus mass prior to embolectomy (Fig 1). If vacuum is lost and/or thrombus mass is retrieved, the aspiration catheter is then again advanced to the occlusion site and a SAVE maneuver is performed by removing both the stent retriever and the vacuum-locked aspiration catheter by additionally aspirating through the balloon-guide/guide catheter. If vacuum still persists after retraction of the aspiration catheter for some centimeters, we continue to pull the aspiration catheter until dynamic vacuum is lost or the aspiration catheter with the clot is completely removed out of the patient. Prior to performing an mSAVE maneuver, the operator studies the CTA carefully (ideally a multiphase CTA [11,13]) in order to be aware of the large clot burden and also the anatomy/position of the posterior communicating artery and anterior cerebral artery. We generally avoid riding the aspiration catheter past the origin of a fetal posterior communicating artery or prominent anterior cerebral artery, in order to avoid shearing off clot and having distal emboli during thrombectomy.

## Results

A total of 207 patients with LVO were treated with SAVE over 29 months. Of those, 20 patients received mSAVE as the primary treatment approach. Median age was 79 years (interquartile range (IQR) 66–83) and 13/20 (65%) patients were male. Baseline characteristics are shown in Table 1. Median baseline NIHSS was 15 (IQR 14–16) and concomitant IVT was applied in 10/20 (50%) patients. Occlusion sites were as follows: ICA in 11/20 (55%), M1 in 8/20 (40%), and basilar artery in 1/20 (5%) patients. All patients presented with a pre-treatment eTICI score of 0. Median Alberta Stroke Program Early CT score (ASPECTS) was 8 (IQR 7–9). Median CBS was 4 (IQR 2–5) and median clot length was 18 mm (IQR 15–25). Individual patient data in displayed in Table 2.

**Table 1:** Baseline characteristics.

Number of patients	20
Baseline characteristics	
Hypertension, n (%)	12 (60)
Coronary artery disease, n (%)	4 (20)
Atrial fibrillation, n (%)	11 (55)
Diabetes mellitus, n (%)	5 (25)
PAOD, n (%)	4 (20)
Chronic kidney disease, n (%)	6 (30)
Obesity, n (%)	4 (20)
Smoker, n (%)	6 (30)

PAOD Peripheral Arterial Occlusive Disease

**Table 2:** Overview about imaging, procedural, and clinical data.

Patient	Age	Site occlusion	Pattern of ICA occlusion	CBS	Clot length [mm]	ASPECTS	Baseline NIHSS	IVT	Number of passes	First-pass eTICI	Final eTICI	Groin puncture to reperfusion [minutes]	ICH	mRS 90 days
1	53	Distal ICA	Linear	2	41	7	13	N	1	2c	2c	39	sICH	3
2	82	M1	-	7	3	8	16	Y	1	2c	2c	31	N	1
3	45	Distal ICA	Linear	1	32	9	14	Y	2	0	3	36	N	0
4	74	Distal ICA	Branching	1	16	6	15	N	1	3	3	43	N	4
5	68	M1	-	2	18	10	12	Y	1	3	3	55	N	0
6	83	M1	-	6	18	9	15	N	1	3	3	52	N	2
7	83	BA	-	-	9	-	3	Y	1	3	3	28	N	0
8	68	M1	-	3	17	8	17	Y	1	3	3	28	N	4
9	68	M1	-	6	27	6	16	Y	1	3	3	15	N	6
10	84	Distal ICA	Linear	1	60	5	15	N	2	1	2a	54	SAH	6
11	77	Distal ICA	Branching	4	17	7	24	Y	1	2c	2c	29	N	3
12	50	Distal ICA	Branching	1	21	7	21	N	1	2c	2c	23	N	4
13	88	Distal ICA	Linear	4	21	7	14	Y	1	2b67	2b67	58	N	1
14	82	M1	-	7	6	6	5	N	1	3	3	19	N	0
15	94	Distal ICA	Linear	5	23	8	16	Y	1	3	3	51	N	6
16	59	M1	-	4	11	8	21	N	1	3	3	36	N	3
17	84	Distal ICA	Branching	4	33	9	24	N	1	3	3	20	N	5
18	61	Distal ICA	Branching	5	26	9	8	N	1	3	3	45	N	1
19	82	Distal ICA	Linear	5	13	5	15	N	1	3	3	15	N	6
20	83	M1	-	5	20	9	15	Y	1	3	3	26	N	0

CBS Clot Burden Score; ASPECTS Alberta Stroke Program Early CT Score; NIHSS National Institutes of Health Stroke Scale; IVT Intravenous Thrombolysis; eTICI expanded Thrombolysis in Cerebral Infarction; ICH Intracranial Hemorrhage; mRS modified Rankin Scale; ICA Internal Carotid Artery; BA Basilar Artery; sICH symptomatic Intracranial Hemorrhage; SAH Subarachnoid Hemorrhage

Complete reperfusion (eTICI 3) after one pass was achieved in 13/20 (65%) and in 14/20 (70%) cases on final angiogram. Successful reperfusion (eTICI ≥2c) after one pass was reached in 17/20 (85%) and in 18/20 (90%) patients at the end of the intervention with an average number of 1.1 ± 0.3 attempts. Out of 11 patients with ICA occlusions, 5 (46%) were reperfused completely and 8 (73%) successfully after a single pass. Nine out of eleven (82%) ICA occlusions were successfully reperfused at the end of the procedure. Two patients with ICA occlusion required a second maneuver due to insufficient reperfusion after the first pass; of those, one patient demonstrated a complete reperfusion result after the second pass, the other patient exhibited an eTICI 2a result. For the latter patient the operator decided to stop the procedure as this was a drip and ship patient with a prolonged time interval from symptom onset to groin puncture of 344 minutes, a low pre-treatment ASPECT score of 5 and difficult access to the supraaortic vessels.

The overall median time from groin puncture to reperfusion was 33 minutes (IQR 25–46). No event of embolization to a new territory (ENT) was observed. One patient suffered from sICH and one patient demonstrated a small subarachnoid hemorrhage (SAH) as demonstrated on FDCT after the intervention. A favorable clinical outcome was achieved in 9/20 (45%) patients at discharge and after 90 days, respectively.

## Discussion

Since endovascular therapy is standard of care in AIS patients suffering from intracranial LVO, existing embolectomy techniques were ameliorated and new methods were developed over the last years. The central requirements of embolectomy comprise a high efficacy including a fast recanalization of the occlusive lesion with a complete reperfusion of the affected downstream territory and a high safety profile including the prevention of ENT and intracranial hemorrhage. Taking these parameters as a benchmark, we recently were able to confirm our results of the SAVE technique with high rates of first-pass complete (modified TICI 3, 45%) and overall complete (56%) reperfusion rates in LVO of the anterior circulation [9]. However, as this technique seemed to be less effective in ICA occlusions compared to M1 occlusions, we modified our approach (so-called “mSAVE”) with the intention to increase probability of reperfusion success in patients with high amount of thrombus as this is often the case in ICA occlusions [7]. In this study, we demonstrate our first experience with mSAVE as a feasible method which seems to be safe and effective.

Debulking of thrombus prior to the final intracranial recanalization procedure was previously described by Eesa et al., who applied manual aspiration through a balloon-tipped guide catheter [14]. The pivotal idea of mSAVE is reducing the amount of clot (Fig 1) by repeatedly retrieving and advancing the aspiration catheter in an alternating fashion, while the catheter is connected with an activated pump and without movement of the stent retriever. The idea is to perform an aspiration maneuver with distal embolus protection, due to the distally placed stent retriever and the remaining distal part of the clot. If vacuum is lost during the retrieval maneuver, the aspiration catheter should be advanced as at this point thrombus has been already retrieved or the contact of the catheter tip to the clot is lost. This step can be theoretically repeated several times, however, in this study it did not exceed four of such alternating maneuvers. While the distally positioned stent retriever acts as an anchor and prevents from clot embolization in the downstream territory, the additional use of a balloon-guide catheter diminishes antegrade flow in the ICA, which may affect the overall complete reperfusion rates. One can assume that the procedural duration could be prolonged by advancing the aspiration catheter several times, however, the median time from groin puncture to reperfusion (33 minutes) was fast. A similar technique was described previously by Mascitelli et al. for cerebral venous thrombosis, who demonstrated that stent anchoring with mobile aspiration technique is effective in a patient with purulent cerebral venous sinus thrombosis [15].

In our study, mSAVE led to a high rate of first-pass reperfusion results (65% eTICI 3, 85% eTICI ≥2c). Especially for patients with ICA occlusions mSAVE seems to be a promising approach as the rate of complete and successful reperfusion after a single pass was 46% and 73%, respectively. In a recent study from Baek et al., only 22% of ICA occlusions were successfully reperfused after one attempt and the rate of overall successful reperfusion (which both were graded as modified TICI) was lower compared to our results (76% vs. 82%) [16]; additionally, in 29% of the successfully reperfused patients four or more passes were necessary. In comparison to our recent results with SAVE, the first-pass and overall complete reperfusion rate is also higher in ICA occlusions (26% and 49%, respectively) [9]. At this point it is worth to mention that in our study the eTICI score was used and that only a reperfusion of ≥90% was defined as successfully compared to the modified TICI score in the aforementioned studies with a definition of success if ≥50% of the vascular territory is reperfused. The high rate of complete reperfusion with mSAVE is essential for the clinical outcome of LVO patients with large clot burden, as complete reperfusion mitigates the effects of time delays and the deleterious effect of age [17–19]. Furthermore, it is to point out that in this study the thrombus amount was large throughout our cohort with a median CBS of 4 and a median clot length of 18 mm. In contrast e.g. in the ASTER trial the thrombus amount was lower with a CBS of 7 and a clot length of 13 mm [20]. This emphasizes that the modification of our technique seems reasonable and effective in AIS patients with a higher clot burden. However, it is still a matter of debate if thrombus amount expressed by CBS influences the reperfusion success with common embolectomy techniques. While in a post hoc analysis of the ASTER trial it was shown that successful reperfusion was less frequently achieved in patients with a low CBS, Treurniet et al. found no evidence that CBS modifies the effect of intra-arterial treatment in the Multicenter Randomized Clinical Trial of Endovascular Treatment for Acute Ischemic Stroke in the Netherlands (MR CLEAN) cohort [5,21]. In our opinion, it is reasonable to assume that thrombus volume is an independent factor of successful reperfusion [16].

The safety profile of mSAVE seems to be acceptable with a five percent rate of sICH. The rate of favorable outcome at 90 days was 45% and is comparable to the results of the meta-analysis of the aforementioned RCTs.^3^ In contrast, despite the promising reperfusion success rate, only 3/11 (27%) patients with ICA occlusion achieved an mRS of ≤2. This could be explained by several factors that might impact follow-up outcome such as pre-stroke mRS status (which was not an exclusion criterion in our cohort), prolonged time to reperfusion (especially in drip and ship patients), reduced collateral flow, and age.

A potential drawback might be a trauma of the inner vessel wall by the repeated movement of the large-bore aspiration catheter. However, we did not observe any vessel injuries such as arterial dissections. A further concern with aspiration prior to stent retriever withdrawal might be that it potentially worsens brain ischemia by decreasing collateral inflow in the ischemic parenchyma through flow reversal in collateral vessels. From our point of view this phenomenon is not observed in distal ICA occlusions due to the high clot burden and may be only an issue in proximal middle cerebral artery occlusions. However, we did not observe any unexpectedly large infarcts despite successful reperfusion.

This study has several limitations. First, this is a small and retrospective study with an attendant selection bias. Second, there were no further inclusion criteria as the operator decided independently from imaging criteria to perform mSAVE. Third, our cohort was heterogeneous with regard to the different occlusion sites. Fourth, individual factors, e.g. clot composition might have an impact on the technical success and was not considered. Therefore, our promising results have to be interpreted with caution and should be confirmed in further studies. Furthermore, it should be proven if mSAVE is a feasible technique for patients with severe arterial stenosis of the distal ICA as the movement of the aspiration catheter might be difficult and therefore unfavorable in such patients. With regard to distal ICA occlusions, future studies have to show if mSAVE is of equal value for linear or branching occlusions.

## Conclusion

Embolectomy using mSAVE is feasible and seems to be safe and effective with high rates of first-pass and overall reperfusion. Therefore, mSAVE might be an alternative method especially in patients with ICA occlusions, where clots tend to be of larger size.

## Supporting information

S1 File STROBE checklist. (DOC)

## References

[1] Rha JH, Saver JL. The impact of recanalization on ischemic stroke outcome: A meta-analysis. Stroke. 2007;38:967–73. 10.1161/01.STR.0000258112.14918.24 17272772

[2] Powers WJ, Rabinstein AA, Ackerson T, Adeoye OM, Bambakidis NC, Becker K, et al 2018 Guidelines for the Early Management of Patients With Acute Ischemic Stroke: A Guideline for Healthcare Professionals From the American Heart Association/American Stroke Association. Stroke. 2018;49:e46–e110. 10.1161/STR.0000000000000158 29367334

[3] Goyal M, Menon BK, Van Zwam WH, Dippel DWJ, Mitchell PJ, Demchuk AM, et al Endovascular thrombectomy after large-vessel ischaemic stroke: A meta-analysis of individual patient data from five randomised trials. Lancet. 2016;387:1723–31. 10.1016/S0140-6736(16)00163-X 26898852

[4] Saver JL, Goyal M, Bonafe A, Diener H-C, Levy EI, Pereira VM, et al Stent-Retriever Thrombectomy after Intravenous t-PA vs. t-PA Alone in Stroke. N. Engl. J. Med. 2015;372:2285–95. 10.1056/NEJMoa1415061 25882376

[5] Zhu F, Lapergue B, Kyheng M, Blanc R, Labreuche J, Ben Machaa M, et al Similar Outcomes for Contact Aspiration and Stent Retriever Use According to the Admission Clot Burden Score in ASTER. Stroke. 2018;49:1669–1677. 10.1161/STROKEAHA.118.021120 29880554

[6] Zaidat OO, Castonguay AC, Linfante I, Gupta R, Martin CO, Holloway WE, et al First Pass Effect: A New Measure for Stroke Thrombectomy Devices. Stroke. 2018;49:660–666. 10.1161/STROKEAHA.117.020315 29459390

[7] Kamalian S, Morais LT, Pomerantz SR, Aceves M, Sit SP, Bose A, et al Clot length Distribution and Predictors in Anterior Circulation Stroke: Implications for IA Therapy. Stroke. 2013;44:3553–6. 10.1161/STROKEAHA.113.003079 24105699 PMC3927722

[8] Seker F, Pfaff J, Wolf M, PA R, Nagel S, Schonenberger S, et al Correlation of Thrombectomy Maneuver Count with Recanalization Success and Clinical Outcome in Patients with Ischemic Stroke. Am. J. Neuroradiol. 2017;38:1368–71. 10.3174/ajnr.A5212 28473346 PMC7959895

[9] Maus V, Henkel S, Riabikin A, Riedel C, Behme D, Tsogkas I, et al The SAVE Technique: Large-Scale Experience for Treatment of Intracranial Large Vessel Occlusions. Clin. Neuroradiol. 2018;1–8. 10.1007/s00062-018-0672-6 [Epub ahead of print]. 30027326

[10] Tan IYL, Demchuk AM, Hopyan J, Zhang L, Gladstone D, Wong K, et al CT angiography clot burden score and collateral score: Correlation with clinical and radiologic outcomes in acute middle cerebral artery infarct. Am. J. Neuroradiol. 2009;30:525–31. 10.3174/ajnr.A1408 19147716 PMC7051470

[11] Psychogios M-N, Behme D, Schregel K, Tsogkas I, Maier IL, Leyhe JR, et al One-Stop Management of Acute Stroke Patients: Minimizing Door-to-Reperfusion Times. Stroke. 2017;48:3152–3155. 10.1161/STROKEAHA.117.018077 29018132

[12] Wiesmann M, Brockmann M-A, Heringer S, Müller M, Reich A, Nikoubashman O. Active push deployment technique improves stent/vessel-wall interaction in endovascular treatment of acute stroke with stent retrievers. J. Neurointerv. Surg. 2017;9:253–256. 10.1136/neurintsurg-2016-012322 26975839

[13] Menon BK, Qazi EM, Almekhlafi M, Hahn L, Demchuk AM, Goyal M. Multiphase CT Angiography: A New Tool for the Imaging Triage of Patients with Acute Ischemic Stroke. Radiology. 2015;275:510–20. 10.1148/radiol.15142256 25633505

[14] Eesa M, Almekhlafi MA, Mitha AP, Wong JH, Goyal M. Manual aspiration thrombectomy through balloon-tipped guide catheter for rapid clot burden reduction in endovascular therapy for ICA L/T occlusion. Neuroradiology. 2012;54:1261–5. 10.1007/s00234-012-1039-3 22552837

[15] Mascitelli JR, Pain M, Zarzour HK, Baxter P, Ghatan S, Mocco J. Sinus thrombectomy for purulent cerebral venous sinus thrombosis utilizing a novel combination of the Trevo stent retriever and the Penumbra ACE aspiration catheter: The stent anchor with mobile aspiration technique. J. Neurointerv. Surg. 2016;8:e24 10.1136/neurintsurg-2015-011782.rep 26019186

[16] Baek J-H, Yoo J, Song D, Kim YD, Nam HS, Kim BM, et al Predictive value of thrombus volume for recanalization in stent retriever thrombectomy. Sci. Rep. 2017;7:15938 10.1038/s41598-017-16274-9 29162921 PMC5698357

[17] Prabhakaran S, Castonguay AC, Gupta R, Sun CJ, Martin CO, Holloway W, et al Complete reperfusion mitigates influence of treatment time on outcomes after acute stroke. J. Neurointerv. Surg. 2017;9:366–9. 10.1136/neurintsurg-2016-012288 27073195

[18] Mctaggart RA, Moldovan K, Oliver LA, Dibiasio EL, Baird GL, Hemendinger ML, et al Door-in-Door-Out Time at Primary Stroke Centers May Predict Outcome for Emergent Large Vessel Occlusion Patients. Stroke. 2018;49:A135. 10.1161/STROKEAHA.118.021936 30571428

[19] Jayaraman M V, Kishkovich T, Baird GL, Hemendinger ML, Tung EL, Yaghi S, et al Association between age and outcomes following thrombectomy for anterior circulation emergent large vessel occlusion is determined by degree of recanalisation. J. Neurointerv. Surg. 2018;1–6. 10.1136/neurintsurg-2017-013671 [Epub ahead of print] 29858396

[20] Lapergue B, Blanc R, Gory B, Labreuche J, Duhamel A, Marnat G, et al Effect of endovascular contact aspiration vs stent retriever on revascularization in patients with acute ischemic stroke and large vessel occlusion: The ASTER randomized clinical trial. JAMA. 2017;318:443–52. 10.1001/jama.2017.9644 28763550 PMC5817613

[21] Treurniet KM, Yoo AJ, Berkhemer OA, Lingsma HF, Boers AMM, Fransen PSS, et al Clot Burden Score on Baseline Computerized Tomographic Angiography and Intra-Arterial Treatment Effect in Acute Ischemic Stroke. Stroke. 2016;47:2972–8. 10.1161/STROKEAHA.116.014565 27827328

